# Towards Camera-LIDAR Fusion-Based Terrain Modelling for Planetary Surfaces: Review and Analysis

**DOI:** 10.3390/s16111952

**Published:** 2016-11-20

**Authors:** Affan Shaukat, Peter C. Blacker, Conrad Spiteri, Yang Gao

**Affiliations:** Surrey Space Centre, Faculty of Engineering and Physical Sciences, University of Surrey, Guildford GU2 7XH, UK; a.shaukat@surrey.ac.uk (A.S.); p.blacker@surrey.ac.uk (P.C.B.); c.spiteri@surrey.ac.uk (C.S.)

**Keywords:** 3-D reconstruction, terrain modelling, LIDAR-camera fusion, planetary surface perception, hybrid vision systems

## Abstract

In recent decades, terrain modelling and reconstruction techniques have increased research interest in precise short and long distance autonomous navigation, localisation and mapping within field robotics. One of the most challenging applications is in relation to autonomous planetary exploration using mobile robots. Rovers deployed to explore extraterrestrial surfaces are required to perceive and model the environment with little or no intervention from the ground station. Up to date, stereopsis represents the state-of-the art method and can achieve short-distance planetary surface modelling. However, future space missions will require scene reconstruction at greater distance, fidelity and feature complexity, potentially using other sensors like Light Detection And Ranging (LIDAR). LIDAR has been extensively exploited for target detection, identification, and depth estimation in terrestrial robotics, but is still under development to become a viable technology for space robotics. This paper will first review current methods for scene reconstruction and terrain modelling using cameras in planetary robotics and LIDARs in terrestrial robotics; then we will propose camera-LIDAR fusion as a feasible technique to overcome the limitations of either of these individual sensors for planetary exploration. A comprehensive analysis will be presented to demonstrate the advantages of camera-LIDAR fusion in terms of range, fidelity, accuracy and computation.

## 1. Introduction

Robotic platforms have experienced a tremendous growth in their usage over the past five decades of planetary exploration missions spanning a diversity of technologies, such as: orbiting spacecrafts [1], space telescopes [2], stationary landers [3,4], etc. However, planetary rovers form one of the most important sources of exploratory information because of their higher degree of mobility, ability of physical experimentation, autonomous navigation and microscopic level of observations. Over the past few decades planetary rovers have evolved into highly complex intelligent systems utilising a variety of onboard sensors that complement its autonomous capabilities. In particular, complex vision systems along with their onboard softwares have played an important role in increasing autonomous capabilities [5].

The National Aeronautics and Space Administration—NASA’s Mars rover Curiosity pioneered the autonomous selection of rock targets for scientific analysis by its laser and telescopic camera suite: Chemistry and Camera (ChemCam), using the Autonomous Exploration for Gathering Increased Science (AEGIS) software [6]. This is just one of the many capabilities that complex vision systems provide on board planetary rovers. In fact, onboard vision systems have become critical components for rover autonomy carrying out complex tasks, for example; high level surface mapping and relative localisation using topological vision data, low level visual feature detection, recognition and landmark tracking, complex scientific procedures such as identification of the chemical compositions of Martian soil, etc. The current work will focus more specifically on topological surface mapping and perception on board rovers, significantly used by other parts of the rover’s Guidance, Navigation and Control System (GNC) [7,8].

The majority of the rover onboard vision systems in past and current missions (such as, Mars Exploration Rover (MER), Mars Science Laboratory (MSL) and Exobiology on Mars (ExoMars)) use cameras for terrain perception, such as; stereopsis for autonomous rover navigation (e.g., visual odometry), hazard detection (e.g., slip perception) and scientific study (e.g., MSL ChemCam) [5]. None of these missions consider loop closures, therefore autonomous navigation to-date is a one-way traverse [5]. However, future missions such as, Mars Sample Return (MSR) would require revisiting previously known landmarks multiple times while travelling much greater distances at higher speeds. Therefore, the use of sensors and techniques beyond standard cameras, stereo vision and stereopsis will be needed for more saturated scene reconstruction covering greater surface area, higher fidelity and feature complexity and more accurate scene perception.

Remote sensing comprising target detection, identification, and depth estimation using Light Detection And Ranging (LIDAR) is commonly used by terrestrial rovers covering very long distances. For space missions, LIDAR technology is mostly used for assisting spacecraft with rendezvous and docking. It seems inevitable that LIDAR systems will eventually form an important part of the rover onboard vision complex. This paper will provide an in-depth review of current methods for scene reconstruction and terrain modelling using cameras in planetary robotics and LIDAR sensors in terrestrial robotics and later propose a camera-LIDAR fusion technique that may overcome the limitations of either of these individual sensors, with potential for use in planetary robotic systems. Comprehensive analysis will be presented to demonstrate the advantages of camera-LIDAR fusion in terms of range, fidelity, accuracy and computation.

The rest of the paper is structured as follows: Section 2 provides a literature survey on current state of the art in terrain perception and scene reconstruction used on board planetary rovers or potentially useful for future missions using cameras and LIDAR technologies. Section 3 introduces the concept of camera-LIDAR fusion as a feasible solution for long distance autonomous planetary rover navigation using a one-shot 3-D reconstruction technique. This is followed by a discussion on all techniques stated in this article in Section 4 to summarise their potential, strengths and weaknesses. Finally, Section 5 presents some important points that require attention for future follow-up based on the current research findings.

## 2. Terrain Perception Onboard Planetary Rovers

Vision-based terrain perception is crucial for planetary rovers allowing them to carry out autonomous and semi-autonomous tasks critical to the success of a mission. Information retrieved from onboard cameras is used for path planning and localisation, hazard detection and avoidance, surface mapping, geological and scientific analysis [5]. Such methods may be distinguished on the basis of the type of sensors used to capture scene data. In the following, each of these techniques will be reviewed in detail to allow a qualitative assessment of their pros and cons in the context of autonomous planetary rover missions.

### 2.1. Cameras

NASA’s Mars Pathfinder mission remains one of the earliest examples of vision-based terrain perception on a remote planet. Operators on Earth used extremely high quality terrain maps of the area around the lander, generated by Jet Propulsion Laboratory—JPL’s real-time stereo algorithm [5] from a multispectral stereo camera pair (installed on the lander) for mission planning. The rover (Sojourner) used a simple light-stripe sensor (measuring 25 elevation points) for its 3-D perception system using the technique discussed in [9]. Since then, stereo vision has been considered as the baseline method for scene reconstruction and perception for planetary rovers.

Stereopsis allows depth perception and scene reconstruction through the use of binocular disparity between visual stimuli from two different sources in the form of either a depth map or 3-D point cloud. The MER rovers (i.e., Spirit and Opportunity) were designed to operate over longer distances (up to 100 m/day) as compared to Sojourner, therefore robust navigation capabilities (such as obstacle detection and avoidance) were deemed imperative. A major evolution was to consider the enhancement of the 3-D terrain perception by using stereo vision instead of laser rangefinders.

**Figure 1:**
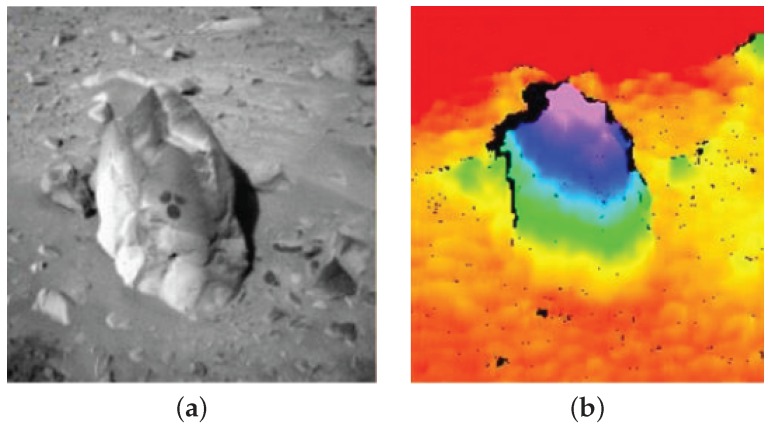
JPL stereo vision image processing on board MER rovers. (**a**) is from the stereo pair, while (**b**) is the elevation map generated using stereopsis (Courtesy NASA/JPL-Caltech).

The MER rovers vision-based obstacle avoidance system used stereo area correlation also known as signal matching. Disparity estimation between two images was computed using Sum of Absolute Differences (SAD) scores [10]. Resolution of the raw 1024×1024 images from the stereo cameras is reduced to 256×256 and rectified with bilinear interpolation and then highpass filtered. Disparity values are computed for each pixel using 7×7 SAD scores followed by subpixel disparity computation via curve fitting. Unreliable disparity values are filtered out along with other noisy artifacts to generate the final output [5]. Figure 1 illustrates an example of the MER rovers stereo image processing. The rovers were also equipped with visual odometry (VO) capability in order to provide support in situations of wheel slippage, absence of global position system (GPS) and absolute positional landmarks [5]. The VO algorithm based on the work by [11,12,13,14,15] takes visual inputs from the existing onboard cameras for the selection, matching and tracking of point features in stereo with multi-resolution correlation to estimate motion between subsequent stereo frames [5]. The MER vision system [16] has provided sufficient basis for the follow-up Mars missions, such as the imaging software on the MSL rover (Curiosity),which has relatively advanced image processing capabilities (e.g., manual and autoexposure, histogram generation, spatial downsampling, spatial subframing, shutter subtraction, bad pixel correction, stereo processing, image metadata collection, etc.) and is set to explore over a range of 5 by 20 km [17].

The ExoMars project is a joint mission of the European Space Agency (ESA) and Russian Federal Space Agency (Roscosmos) due to be delivered to the Martian surface in 2020. It is designed to carry a range of complex vision-based sensors for environmental/terrain perception and scientific study. The ExoMars rover perception system uses a pair of stereo images to generate a disparity map. The onboard processing is quite similar to the MER and MSL rovers 3-D perception system. The stereo images are rectified within the cameras and downsampled to different resolutions for separate sections of the disparity map. Gradient images are produced using Laplacian of Gaussian (LoG) pre-processing filter prior to the application of the stereo-correlation algorithms. A SAD algorithm is then applied to generate a disparity map followed by linear sub-pixel interpolation to calculate decimal disparity. Erroneous values are removed from the raw disparity map to produce the final output [18]. The use of variable resolutions for different depth of fields reduces processing load under very limited onboard computational resources.

The lunar rover Yutu (“Jade rabbit”) is the first ever Chinese rover deployed on the lunar surface as a part of the China National Space Administration (CNSA) unmanned lunar exploration mission Chang’E-3 [19]. The onboard vision payload consists of two panoramic cameras and two navigation cameras on the rover’s mast and two hazard avoidance cameras on the front side of the rover, each capable of producing stereoscopic images that can be used for depth estimation and navigation via stereopsis.

**Figure 2:**
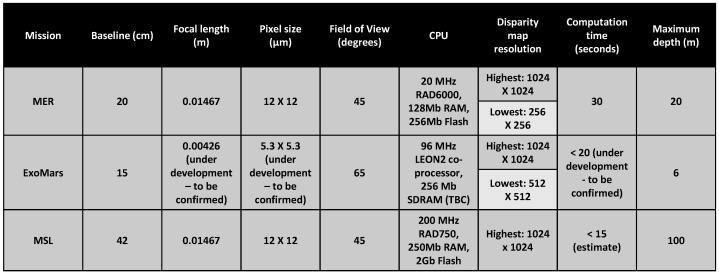
Comparison of the stereo depth perception systems on board MSL, MER, ExoMars rovers.

**Figure 3:**
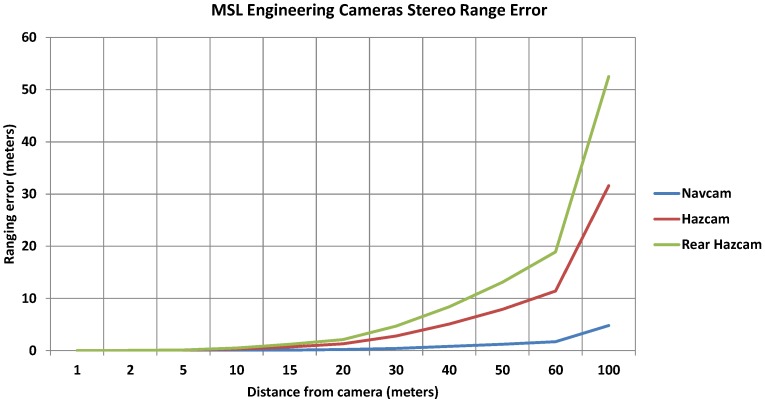
MSL engineering cameras stereo range error as a function of distance from the camera.

Decades of planetary rover missions have verified, tested and established the feasibility of stereo cameras and stereopsis as the sole 3-D perception technology onboard. It has the advantage of being solid-state and hence mechanically robust and longer lasting. Furthermore, research findings have shown that Martian terrain has enough textures for stereo vision to be applied almost anywhere. Algorithms that can perform stereopsis and produce sufficiently dense and accurate range imagery at sufficient speed using the available computing resources are well established. It has therefore been considered as the best trade-off between cost, risk, and performance for 3-D terrain perception on board planetary rovers [5]. Figure 2 presents some features and performance measures of the 3-D perception systems on board different planetary rovers [17,20]. Figure 3 presents plots of the stereo range error for multiple engineering cameras on board the MSL rover based on literature from [17]. There is a significant decrease in the accuracy of the estimated depth with the increase in distance from the camera, which may not be deemed suitable for long range terrain modelling and perception. There is also an increase in the computation complexity if higher resolution cameras are used. Hence stereopsis does not provide an optimal solution beyond a few metres from the rover for future more challenging planetary rover missions (e.g., MSR) [8], which will require highly accurate scene reconstruction for absolute localisation.

There is an increasing understanding that methods based on monocular vision may offer greater potential for terrain modelling and perception for planetary rovers [7]. Monocular image processing may have a lower computational demand as compared to stereo vision processing and is able to detect objects at a much further distance. However, accurate 3-D reconstruction of the terrain from monocular images is a very challenging problem [7]. Although recovering depth from single monocular images is an extensively studied research topic within the scientific community [21,22,23,24,25]; they fail to provide feasible solutions for planetary rovers because of the algorithmic complexity and required computational resources. For example structure from motion is a well known technique for the reconstruction of large uncontrolled environments using monocular cameras in standard 3-D computer vision applications. However time complexity of incremental structure from motion is often stated as $O(n^{4})$ [26], which is beyond current state-of-the art space qualified processors. Supervised learning techniques have also been applied to monocular images for dense 3-D reconstruction [22]. A probabilistic model such as the Gaussian Markov Random Field (MRF) is used to capture multiscale depth relations using a training set of natural unstructured indoor and outdoor environments (e.g., forests, trees and buildings etc.), and the corresponding ground-truth depth maps [22]. This technique may fit well for terrestrial applications due to unlimited access to data with frequently occurring natural scenes and computing resources, but it may completely fail to capture the surface characteristics of Mars due to limited datasets and processing resources.

**Figure 4:**
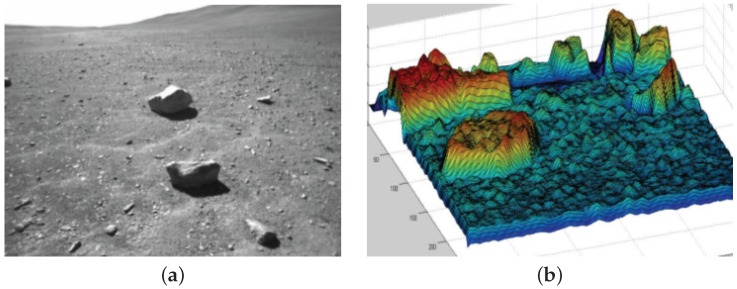
Image of a representative planetary surface (**a**); DTM generated using SfS method (**b**).

Photoclinometry or shape from shading (SfS) techniques are used for estimating topography mostly using a single monocular camera. It has been a topic of research interest within the planetary science community for more than 50 years [27]. The techniques are classified as global and local methods. Global methods involve minimising a cost function in order to recover the surface depth through variation calculus and tend to be more complex. However, the results produced are better than local methods. Local methods are simpler but only provide an approximation of the 3-D shape [28,29]. The SfS reconstruction process assumes the reflectance map is known a priori and the albedo map is homogeneous. For terrestrial applications homogeneity of albedos may not always be the case due to non-linearity in natural scenes. However, surfaces on the Moon and Mars tend to have a restricted range of albedos with distinct variations that can be easily detected [28,29]. Furthermore, since the technique relies on the direction of the light source, for example the sun, this can be easily deduced from the rover’s onboard sun sensor. All of these characteristics make it an ideal method for reconstructing the visual scene environment. In fact, recently this technique has been applied to high resolution images from Chang’E-3 (CE-3) lunar mission for lunar soil physical properties analysis [29] and object detection of planetary surficial rocks for autonomous navigation and mapping [28]. Figure 4 illustrates an example of a digital terrain model (DTM) generated using SfS technique.

Most monocular 3-D reconstruction techniques have only been tested as proof-of-concept for planetary rover applications. Generally, a major disadvantage with such techniques is that their accuracy and performance critically depends upon additional sources of information (e.g., wheel odometry, inertial measurement units, etc.), which may require using some type of state estimation filters (e.g., Kalman filtering) [30]. Metric measurements becomes impossible without such additional data. Furthermore, such filtering techniques aren’t optimised for space qualified processors [8].

### 2.2. Light Detection And Ranging (LIDAR)

**Figure 5:**
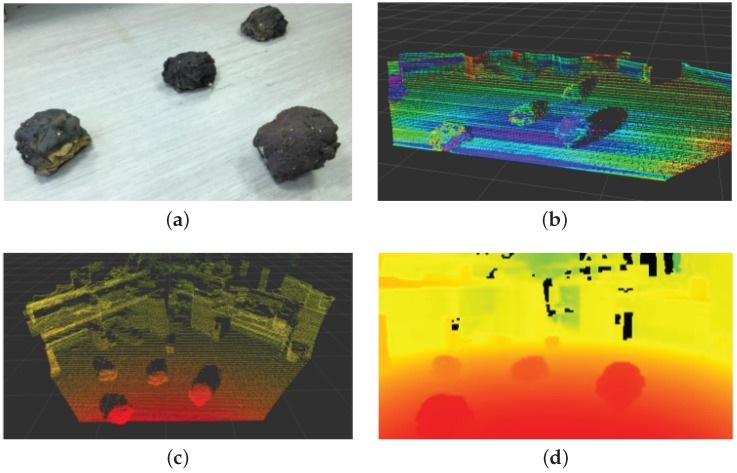
Example illustration of a scattering of LIDAR points (**b**) of a flat surface with rock boulders (**a**); point cloud (**c**) and depth map (**d**).

Light Detection And Ranging (LIDAR) technology has been used extensively for precise long and short target detection, identification, and depth estimation. It is broadly classified into three major types: range finders (environmental perception, depth estimation and digital elevation models, etc.), Differential Absorption Lidar (DiAL; for measurement of atmospheric temperature and trace gases pressure etc.), and Doppler LIDAR (velocity measurement) [31]. It uses time-of-flight principles to get depth measurements of the surrounding environment that can be effectively used for 3-D surface mapping and scene reconstruction, refer to Figure 5 for an example illustration. For space applications, LIDAR is used for spacecraft assistance with rendezvous and docking, depth estimation and mapping, scientific analysis and geological surveying. For example, the OSIRIS-REx spacecraft (Lockheed Martin, Bethesda, MD, USA) (planned for launch in 2016) will attempt to map the asteroid 1999 RQ36 using the OSIRIS-REx Laser Altimeter (OLA) (Canadian Space Agency, Quebec, QC, Canada). An advance version of the Laser Camera System by Neptec Design Group; TriDAR (Triangulation and LIDAR Automated Rendezvous and Docking system) was tested on the STS-128 , STS-131 and STS-135 missions to track the International Space Station (ISS) during flight operations and is undergoing further development as a navigation system for lunar rovers [31].

Many techniques involving point clouds generated by 3-D laser rangefinders have been used for topographic mapping of planetary surfaces. However, these techniques have been tested on terrestrial Mars-like test facilities with the potential for future planetary rover missions. The authors in [32] proposed a novel approach towards improving the convergence properties of scan registration algorithms by using curvelet transforms for topographic mapping. The technique has been evaluated using simulated scans from Neptec Design Group’s IVIGMS 3-D laser rangefinder against existing techniques and manifested better performance under standard complexities in planetary analogue terrains such as occlusions and weak geometric features. LIDAR has been used for generating digital elevation models (DEM) to detect slope hazards at longer distances. A sound example of one such implementation is described in [33], with a broader scope for fractal-based lunar surface modelling. A DEM is generated using LIDAR with a 10×10 degree field of view, 100×100 pixel resolution, and 1 Hz scanning frequency. Another LIDAR based approach introduced in [34] uses 3-D scans of the ground surface originating from a planar LIDAR ($180^{\circ }$, $0.5^{\circ }$ angular resolution) fitted to a panning mechanism on board the rover. The plane of scanning ray is orthogonal to the ground plane with an effective range of approximately 20 m. Each of the vertical scan lines is processed to generate a map of the terrain. Perceived terrain characteristics are then used to identify slope hazards. Other methods using LIDAR scans for terrain mapping can also be found in [35,36,37].

In order to make LIDAR technology more feasible for planetary rovers, researchers have also focused on improving the hardware technology itself apart from software post-processing. The Compact Fast Scanning Lidar (CFSL) was developed by MDA and Optech as a compact active sensor for terrestrial and space vehicle navigation applications and was found to perform better than most of the state-of-the-art technologies in terms of resolution, scan speed, field-of-view and high data output rates [38]. Performance of the prototype system has been demonstrated in indoor and outdoor environments for a number of planetary rover applications. The Canadian Space Agency (CSA) tested a commercial LIDAR sensor: an ILRIS-3D sensor from Optech for a 2.5-D point cloud generation of the terrain as a potential solution for long distance (over-the-horizon) autonomous planetary rover navigation [39]. Similar work has also been carried out in [40] using range data from LIDAR sensors and converting it into Irregular Triangular Meshes (ITMs) for an accurate representation of the environment. The proposed technique has been extensively tested and validated in an autonomous navigation scenario at the CSA’s Mars emulation terrain (MET).

These techniques using LIDAR as a potential technology for planetary rover navigation helped identify the intrinsic limitations with these active sensors. First and foremost is the time and computational resources required to capture and process full $360^{\circ }$ high-resolution scans [41]. Furthermore, LIDAR data points can have artifacts such as laser shadowing due to surfaces without a clear line-of-sight, obstacles, occlusions or obstructions. Semantically labelling a cluster of LIDAR points related to an object of interest can prove to be a very difficult task in terms of computer vision applications [41]. It is therefore important to investigate methods that can potentially improve current LIDAR-based environmental perception and scene reconstruction solutions for future planetary rover missions.

## 3. Camera-LIDAR Fusion for Planetary Terrain Perception

Precise mapping of visually perceived information from camera onto LIDAR data for terrain modelling and reconstruction combines the advantages of both sensors by having saturated texture features alongside highly accurate depth information that may span over hundreds of metres. Ideally, 3-D LIDAR technologies are well adapted for such applications. However, relatively lighter and cheaper 2-D laser range finders have also been used for 3-D scene reconstruction of indoor and outdoor scenes with the help of actuators and pan/tilt units [42,43]. However, the task of correctly collating RGB data from camera with three dimensional point cloud data from a LIDAR sensor is computationally complex and non-trivial. Firstly, raw point cloud from most LIDAR sensors may contain sporadic missing depth points due to varying reflectance, absorption and geometric properties of the incident surfaces and the relative distance between the target surface and the sensor. Hence, it can not be used directly without the necessary post-processing. Secondly, colour images from a camera may have optical artifacts or distortion in the absence of accurate intrinsic calibration. Moreover, LIDAR data can be extremely dense and become extremely complex in terms of computational load for rover onboard processing. Hence, most techniques in literature are well suited for terrestrial applications [42,43,44], fail to address some critical constraints related to planetary rover applications, such as; computation cost and memory usage without compromising accuracy and fidelity of the reconstructed surface or terrain. In the following, a method for combining LIDAR data with visual information from camera will be investigated in the context of planetary rover missions using a one-shot 3-D reconstruction technique. The output of this hybrid sensing method will be a 3-D reconstruction of the surrounding environment in the form of surface meshes. A major objective of this work will be to maintain very low computational complexity, memory usage and error, whilst adding actual texture information of the visual scene from the camera instead of adding false colour information. This can potentially be very useful for geological and scientific study in addition to merely navigation (such as [41]). Furthermore, it may also be used for the purpose of matching orthographic ”top-down” view of the scene reconstruction with existing satellite imagery, which requires colour and intensity information in addition to 3-D topology as described in [45]. Success of achieving these objectives will be tested and analysed in varying indoor and outdoor experimental environments using the SMART robotic platform developed by the STAR lab [30].

### 3.1. Hardware Setup

**Figure 6:**
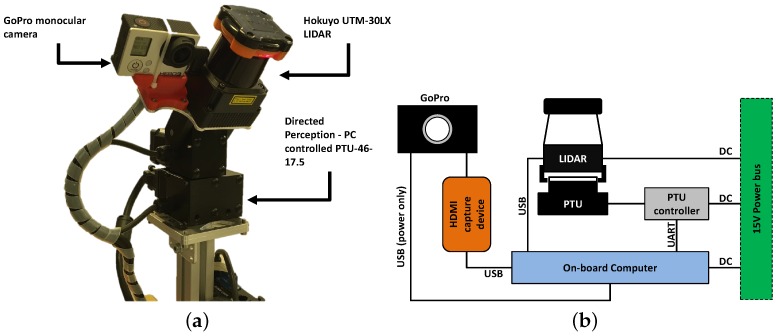
Proposed hardware setup for fusion of camera and LIDAR data for planetary scene reconstruction (**a**); and device connections (**b**).

The current hardware setup is presented in Figure 6. It consists of a wide angle lens camera; a GoPro Hero 3 with $120^{\circ }$ horizontal field of view (H-FOV), and $90^{\circ }$ vertical field of view (V-FOV), fitted close to a Hokuyo UTM-30LX 2-D LIDAR. Both sensors are fixed to a common metallic panel actuated by a Directed Perception PTU-46-17.5 Pan-Tilt unit (PTU). The baseline between the camera and the LIDAR is fixed to 43 mm. The use of a wide angle lens camera mitigates the requirement of capturing several narrow FOV images in succession to cover the whole length of the LIDAR FOV, thus saving onboard memory and computational resources that may be required for post-processing, e.g., image stitching. Each individual perception sensor is controlled via various program executables implemented using the Robot Operating System (ROS). Data is captured from the LIDAR sensor for the full width and height of the camera’s FOVs. The camera’s V-FOV is wider than the maximum tilt of the PTU, therefore it is limited by its range. The PTU has a maximum pan range of $\pm 160^{\circ }$, full $360^{\circ }$ panoramic scans can be produced by repeatedly recording multiple scans and capturing camera images at varying pan angles and stiching them together.

### 3.2. Sensor Data

**Figure 7:**
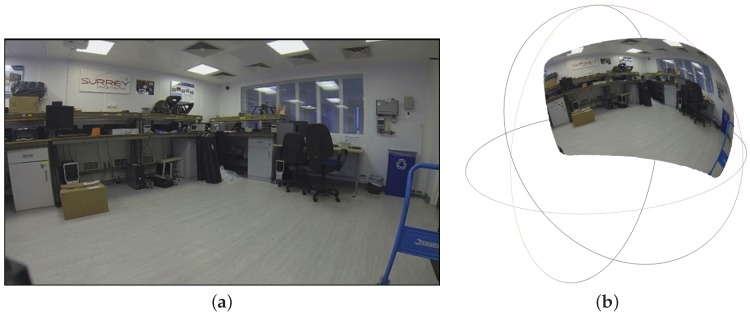
Image of an indoor laboratory environment illustrated as a flat array (**a**); and projected onto a unit sphere using its intrinsic lens model (**b**).

The frames from the camera are captured at a resolution of 1280×720 pixels in the form of RGB intensity images. A 3-D point within the field of view of the camera and the image plane are related via the camera’s lens model. Although a number of such models exist in literature, the simplest and most commonly used is the pin-hole projection model that fits well for rectilinear lenses [46]. However, the more complex Brown-Conrady model fits the current wide-angled GoPro camera lens with spherical distortion [47], which has been considered a feasible solution for the current hardware setup. However, other models, such as [48] can be found in literature, which may suit a greater range of wide-angle and fish-eye lenses. Figure 7 illustrates the projection of the image from the camera sensor onto a unit sphere using the Brown-Conrady model. The use of this model provides a mathematically convenient description of the direction of the incident light rays entering the camera lens. The geometric meaning of an array of image pixels can then be defined by the mathematical relationship between the locations on the camera image and the surface of a unit sphere.

**Figure 8:**
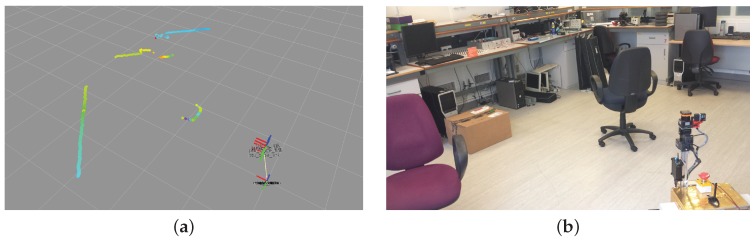
Single line of LIDAR data points (**a**) of the indoor laboratory setup (**b**).

**Figure 9:**
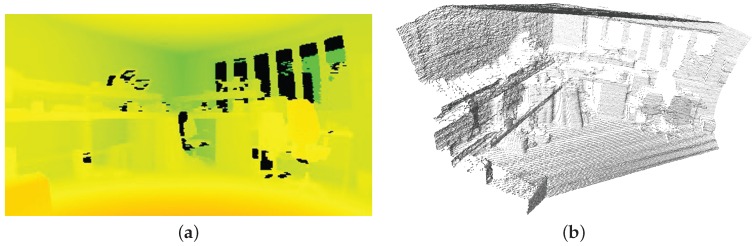
Depth map generated from LIDAR points (**a**); and the reconstructed 3-D point cloud (**b**).

**Figure 10:**
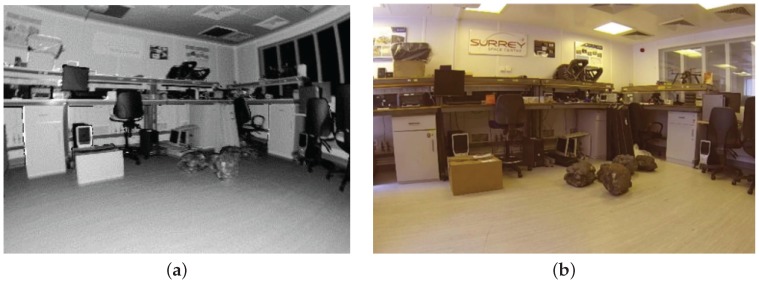
Image produced from LIDAR intensity measurements (**a**) of the laboratory setup (**b**).

The Hokuyo UTM-30LX LIDAR consists of a mirror rotating at 2400 RPM and a $270^{\circ }$ field of view, $0.25^{\circ }$ angular resolution, producing a full scan of LIDAR measurements at 40 Hz at a maximum depth of 30 m. Sensor measurement resolution is 1 mm, and sensor accuracy is 0.1–10 m: ±30 mm, 10–30 m: ±50 mm. Figure 8 illustrates a single line of 3-D points from the LIDAR sensor relative to the global location of the sensor and rover. The high speed scanning mechanism of the LIDAR sensors allows the use of a simple tilting mechanism at relatively lower frequency to record 2-D grids of LIDAR points. Using the depth information from the LIDAR sensor and the precise horizontal beam and vertical tilt angles per sample; the captured data can be used to generate an array of 3-D points as shown in Figure 9. In addition to depth information, the LIDAR sensor also measures the intensity of the reflected laser beam, which is very useful for both planetary rovers and the sensor fusion itself. This measurement is completely independent of ambient light sources and represents the reflectivity of the surface to the frequency spectrum of the laser. The LIDAR used in the current setup incorporates a 905 nm laser in the near-infrared region (NIR) of the electromagnetic spectrum, hence it is a measure of the NIR reflectivity of the surrounding environment. Referring to Figure 10, the LIDAR intensity image manifests a unique response to a different region of the optical spectrum than the camera, without shadows or hotspots. This type of active sensing-based visual perception provides a more consistent intensity image as compared to the camera, which is dependent on ambient light sources, especially in variable uncontrolled extraterrestrial planetary environments. There will be sporadic data points with spurious values in the intensity image due to the reflectance of specific surfaces, or regions with surface anomalies.

### 3.3. Sensor Calibration

**Figure 11:**
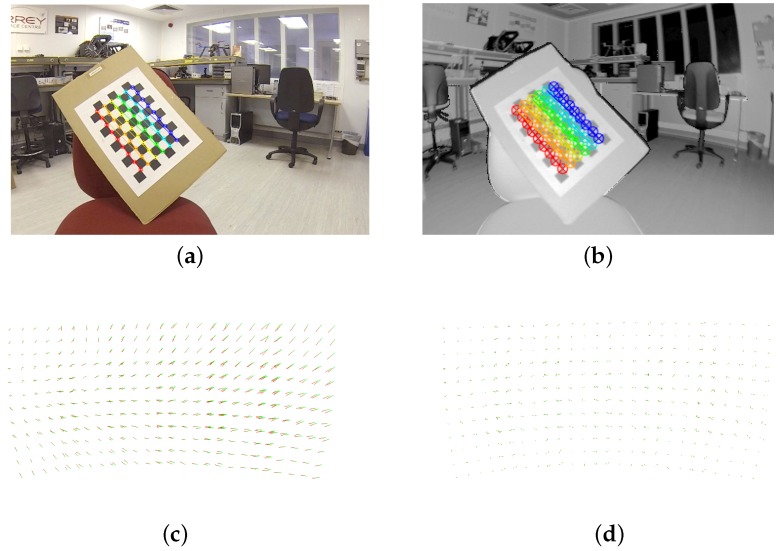
Extrinsic and intrinsic calibration of the sensor suite, (**a**,**b**) illustrate the camera and LIDAR views respectively; (**c**,**d**) illustrate the target points before and after calibration respectively.

The calibration system is used to tune both the extrinsic transformation between the camera and the LIDAR as well as the parameters of the lens model used. A weighted gradient descent algorithm [49] is used to simultaneously refine these parameters based on a quality metric. Exploiting the standard 2-D plane-based camera calibration technique [50]; rotationally asymmetric checker board patterns are used which can be detected in the camera image and the NIR image from the LIDAR as shown in Figure 11. The position of each checker board corner can be precisely determined in both sensors’ space, resulting in a set of positional pairs. The positions in the LIDAR data represent 3-D points, which are projected into the camera image frame using the fusion function. The distance between the detected camera point and the projected LIDAR point is a measure of the calibration error present in the system. The sum of squares of the set of these distances is the quality metric used by the gradient descent algorithm. This calibration method was used to reduce the mean calibration error to less than the size of a LIDAR sample, which is 2.6 (camera) pixels. Sets of points before and after calibration are illustrated in Figure 11.

### 3.4. Sensor Fusion for Surface Reconstruction

The process of fusing camera and LIDAR data using the geometric relationship between both data types results in a more saturated source of information than provided by either of the individual sensors. The resulting output may contain full colour texture information with precise 3-D topology of the surface. In the following, two principal methodologies to present this fused data will be reviewed with a discussion of why the more complex meshed surface is chosen for the current application domain.

**Figure 12:**
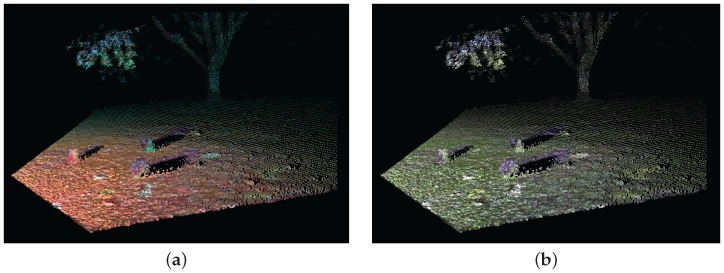
Point cloud of camera-LIDAR fusion showing the RGB intensity values of each 3-D point (**b**); and a multispectral view of the point cloud showing R, G and NIR channels (**a**).

The simplest method of combining camera inputs with LIDAR data is to map each 3-D LIDAR point onto a pixel in the camera image frame, thus associating RGB colour intensity values from the camera to each sample in the point cloud. The output from this process is the same as the LIDAR point cloud with three additional channels of intensity values, that is; red, green and blue for each valid sample. Furthermore if this data is combined with the existing infrared intensity information, it produces a multi-spectral 3-D data. Figure 12 illustrates examples of these different combinations of visual channels.

Using the Brown-Conrady camera model [47], 3-D points are projected into the image plane as a set of 2-D pixel positions via perspective transformation. Most of these pixel positions are within the camera frame; however some may fall outside the image if the field of view of the LIDAR is greater than that of the camera. Similarly, it is also possible that regions of the camera image may lie outside the LIDAR’s field of view. The current sensor setup comprises a very wide angle monocular camera and a scanning LIDAR system where the field of view can be controlled. The field of view of the LIDAR was chosen so that it was entirely contained by the camera’s field of view, and only the samples within the overlapping regions of both sensors were processed.

There are a number of limitations related to this method. The resolution of the camera image will generally be much higher than the resolution of the LIDAR depth map. Given the current experimental setup with the LIDAR resolution at 320×260 samples and the camera resolution at 1280×720 pixels, the camera has approximately 11 times higher resolution than the LIDAR. As such, if the fusion data is represented as a point cloud with the colours extracted from the camera image then the majority of pixels in the camera image will be ignored. Furthermore, an intrinsic problem with point clouds produced by LIDAR in general is the variation in the density of points caused by the distance of a surface from the sensor or the angle between the surface and the direction to the sensor, thus causing unwanted artifacts in the output data.

**Figure 13:**
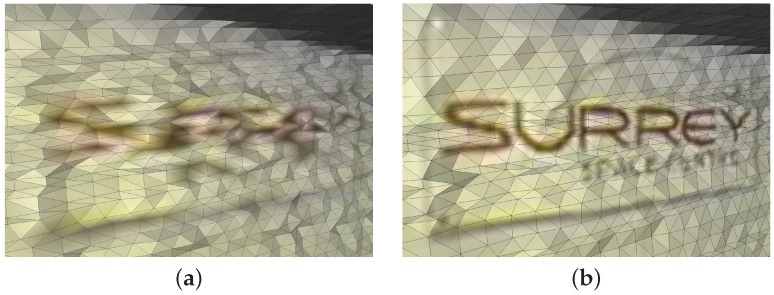
Surface mesh rendered with vertex colour (**a**); and texture mapped with full resolution RGB image (**b**).

Due to the characteristics of extraterrestrial planets deemed safe for planetary rover missions (such as Mars), it is more geometrically accurate to represent it as a surface (mesh) than a collection of 3-D points. Due to the inherent limitations in point cloud data mentioned before, the current setup produces a surface representation of the environment. A triangulated mesh structure represents more information about the scene than a point cloud alone. The triangles that make up a mesh have explicit surface normals, which define the external side of the surface as well as the curvature when combined with the neighbouring triangles. Furthermore, meshes can also be simplified in flat areas, thereby reducing the cardinality of data needed while still accurately reconstructing the planetary surface. If the data output of the fusion process is a texture mapped surface; then the full resolution of the camera data is preserved when it is applied to the 3-D model at lower resolution. Figure 13 illustrates examples of the relative quality of a mesh with colours defined only at vertex locations and with the full resolution camera image texture mapped onto it. A triangulated mesh which is texture mapped with the full resolution camera image is the richest way of representing the fused data, since it preserves the full resolution of all the sensors. Additionally, the lower resolution NIR intensity values from the LIDAR can also be combined with the higher resolution of the camera image.

### 3.5. Point Cloud Triangulation and Surface Mesh Generation

**Figure 14:**
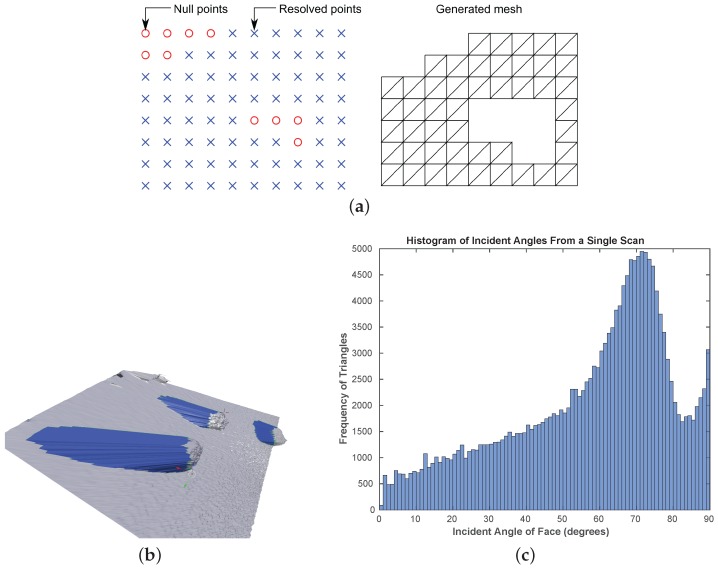
Triangulation of a structured point cloud (**a**); false surface regions shown in blue generated by the mesh triangulation process (**b**); and the histogram-based frequency distribution of the incident angles for a typical scan with large occluded areas (**c**).

The raw output from the LIDAR sensor is a structured point cloud arranged in a rectangular grid including some null points at locations where the sensor was unable to determine the depth. Structured point clouds can be easily triangulated because of the layout of the 3-D points, which mitigates any anomalies caused due to the surface overlapping with itself. The points in structured point clouds have explicit neighbours, therefore a triangular mesh can be created as illustrated in Figure 14a. A pair of triangles are generated for every adjacent set of four non-null points. There are regions within the generated surface between the boundaries of objects at different depths, where false faces are created by the triangulation algorithm. This is illustrated in Figure 14b; showing a triangulated surface joining the edges of the rocks with the edges of the occluded regions on the floor. These false surface regions are identified by calculating the angle (*θ*) between the surface normals and the vector joining the centre of the surface with the LIDAR’s point of view. A triangle directly pointing towards the sensor will have θ=0 and for a region in the plane which passes through the viewpoint $\theta =90^{\circ }$. Using a histogram-based frequency distribution of all the face normals covering the shadowed regions of the surface map, it was empirically found out that rejecting surface regions with $\theta >87^{\circ }$ removed all significant outliers for the current sensor setup (cf. Figure 14c). It is known that the false faces produced by the meshing process will have incident angles close to $\theta =90^{\circ }$, which can be clearly seen to be distinct from the main distribution as outliers.

There are two important factors related to the triangulated meshed surface that requires further attention in the case of planetary rovers. Firstly, a large amount of data is generated. For example, a point cloud made of 130 lines with 320 points per line when triangulated, produces a mesh with 34,500 vertexes and 65,900 triangles. This results in approximately 4.6 megabytes of data, compared to the compressed camera image size of 170 kilobytes. Secondly, the spatial resolution of the point cloud varies from dense to sparse points with the increase in the distance from the sensor.

A system that is capable of reducing the complexity of dense areas of the mesh would reduce the processing requirements of downstream processing, without losing any meaningful information about the terrain topology. Such mesh simplification algorithms can be found in literature. Reviewing the available algorithms [51]; Quadratic Edge Contraction (QEC) developed by Michael Garland and Paul Heckbert [52] was found to be most suitable for the current application. This algorithm is fast and capable of preserving the geometric accuracy of the mesh itself. It works by sequentially merging vertices using a minimised error criterion, and the process continues until the desired vertex count or quality level is reached. Most of these standard mesh simplification algorithms including QEC are designed to work on a full resolution mesh, which is then decimated to a lower resolution as a post-process. Following this procedure would entail the creation of a full resolution mesh and later downsampled using one of these techniques. For the current application, an ideal solution would be to process the LIDAR data per line and generate a lower complexity mesh as the actual output. Thus a full resolution mesh wouldn’t be required, freeing up memory usage and CPU resources. One such technique is proposed below.

### 3.6. Proposed Pipelined Mesh Simplification Algorithm

This method creates a mesh from a structured point cloud at a specified resolution. The input to this algorithm is a sequence of lines of LIDAR points and the output is a triangulated mesh. The mesh is created in stripes with each line of LIDAR points processed in succession. The required quality of mesh is defined by a spatial resolution; *δ* parameter that defines the minimum distance between a new vertex and all previous vertices to be included in the simplified mesh. A smaller value of *δ* results in a denser, higher resolution mesh while a larger value creates a sparser lower resolution mesh.

**Figure 15:**
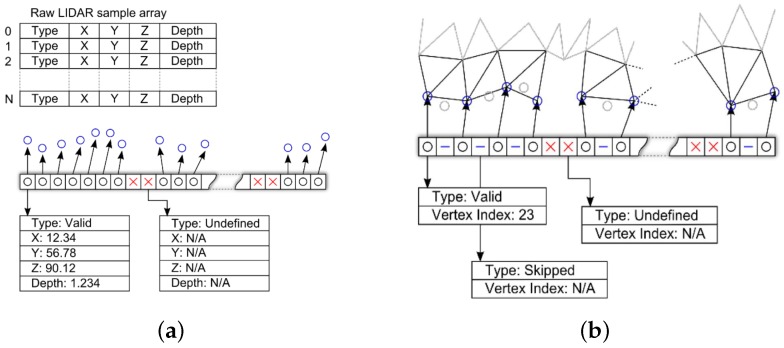
Data structure representing a line of raw LIDAR points (**a**); and the current edge of a mesh being created (**b**).

The data structure used to describe a line of raw LIDAR samples is illustrated in Figure 15a. Each scan line of raw LIDAR samples is a fixed length array containing the type of this sample (valid or undefined), the 3-D position of the sample (if known), along with its depth and intensity. Another data structure temporarily holds a description of the edge of the mesh while it is being created, illustrated in Figure 15b and has the same size as the raw LIDAR data. Each element in this array contains a value which defines the type of that sample, and an optional vertex index if this element is linked to a vertex on the edge of the mesh. Two different methods are used to reduce the vertical and horizontal resolution of the mesh during this process. To reduce the vertical resolution when a new line is processed; the mean Cartesian distance; $\alpha_{v}$, between the vertices of the new line and the old line is calculated and if $\alpha_{v}>\delta$, it is added to the mesh, otherwise it is ignored. To reduce the horizontal resolution the Cartesian distance between the current vertex and the last vertex; $\alpha_{h}$, in the line that was added to the mesh is calculated, and if $\alpha_{h}>\delta$ the vertex is added to the mesh, otherwise ignored.

The proposed algorithm has been designed such that it can process at high speeds a low power space qualified processor. The simplified mesh produced by this process is not always even or mathematically ideal, however it produces meaningful data describing the environment in the field of view of the sensor. In the following, the process of adding each line of raw LIDAR points to the mesh at the required *δ* is described in more detail.

**Figure 16:**
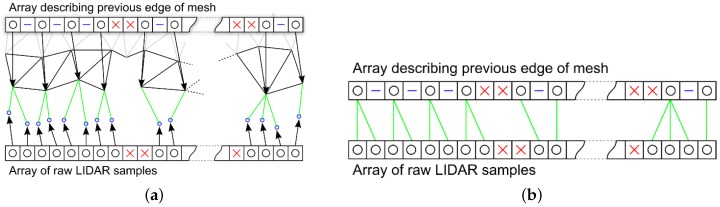
Calculating the mean Cartesian distance between lines (**a**); and between array elements (**b**).

To calculate the mean Cartesian distance between a new line of raw LIDAR samples and the most recent edge of the mesh (Figure 16a); the distances between each valid vertex in the raw LIDAR samples array and the closest vertex in the previous edge array is calculated (Figure 16b). The definition of closest here is based on their position in the respective arrays not the geometric position of the vertices they represent. The mean of all these distances is taken as the value used to test against *δ* to determine if this line needs to be added to the mesh.

**Figure 17:**
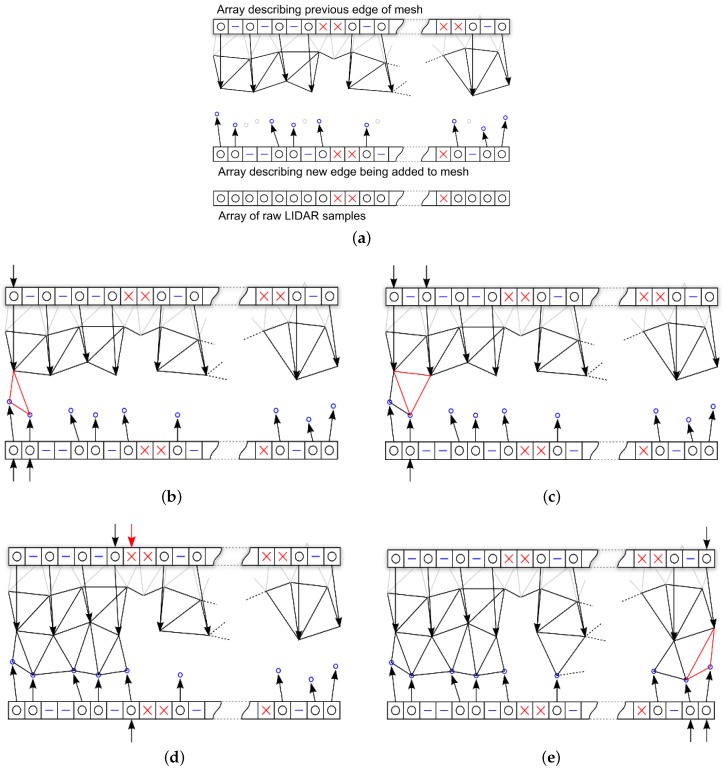
Method used to reduce the resolution of the line of raw LIDAR points and adding to the mesh creating a new edge array (**a**) and process of triangulating a stripe of reduced resolution LIDAR data into the mesh (**b**–**e**).

When a line of raw LIDAR samples is processed, the vertices that qualify are added to the mesh structure and a new edge array is created. Every element of the raw LIDAR samples array is added to the edge array as either a valid, skipped or undefined element. If the sample is valid and added to the mesh, then the edge array will also contain the index of this new vertex in the mesh data structure. Referring to Figure 17a; the rules describing this process are as follows:

- The first and last valid vertices in the raw data array are always added to the mesh and a reference added in the new edge array.
- A valid vertex is added to the mesh and the new edge if its Cartesian distance from the previous is greater than the required *δ*.
- If a vertex is less than *δ* from the previous added vertex then a skipped entry is added to the new edge array.
- Undefined vertices are always added to the new edge array.
- A valid vertex that follows an undefined vertex is always added to the new edge array.

Once a new line of vertices has been determined and added to the mesh structure, triangles need to be added in the appropriate places joining the old and new edges together:

The first three non-skipped vertices are selected; if all three of them are valid a triangle is created using them. Then the group of three selected vertices is moved one step along the stripe to the location of the next potential triangle (Figure 17b). This process is repeated adding triangles to the stripe between each set of three valid vertices (Figure 17c). If any of the three vertices are undefined then a triangle is not created but the process of stepping the three vertexes continues in the same way as before (Figure 17d). When the last set of three vertices have been processed, the triangulation of this line of data is complete. The final step is to replace the previous edge array of vertex information with the new edge and continue with the next line of LIDAR data (Figure 17e).

**Figure 18:**
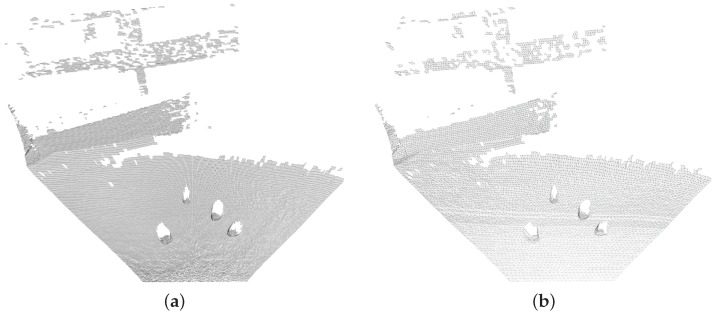
Full resolution mesh with 116,000 triangles (**a**) and simplified mesh (δ=0.1 m) with 13,700 triangles (**b**).

Figure 18 illustrates an example output of this technique, used to generate a surface from an outdoor LIDAR scan with fake rock boulders in the view. The lower resolution mesh carries a lower CPU load and memory occupation during the reconstruction process, hence speeding up downstream processes. Although the simplified mesh will have a lower density of points compared to the full resolution, however the absence of saturated points is augmented by texture mapping of the camera image onto the surface. The resulting surface DTM can potentially be very useful for scientific analysis of the Martian topology by geologists using downlinked data and marking important regions of interest for the Mars sample return missions. More detailed analysis of the output of this algorithm is presented in the following section.

### 3.7. Performance Analysis

**Figure 19:**
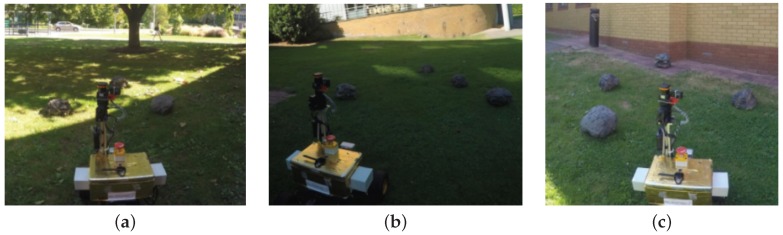
The test sites chosen for performing the experimental analysis; Site A (**a**), Site B (**b**) and Site C (**c**).

The following quantitative analysis of the proposed low-cost planetary surface reconstruction technique will seek to determine the potential advantages in terms of reduced computational complexity and occupation of lower memory resources without significantly compromising on accuracy and fidelity. These experiments have been performed using the Robot Operating System (ROS) in C++ on a mobile workstation with quad-core Intel Core TM i7-4700MQ CPU (2.40 GHz), 16 GB RAM running Linux Ubuntu 14.04 LTS with 64-bit architecture. Three different test sites were chosen to record the data, refer to Figure 19. The images were recorded using the instrumented rover introduced in Section 3.1, representing vegetated surfaces outside the main building. ”Site A” represents an open space region with a scattering of artificial rock boulders and varying light conditions (i.e., shaded and some areas illuminated by direct sunlight), ”Site B” represents an area mostly shaded by buildings, open space and a scattering of rock boulders, and ”Site C” represents a closed region facing a building wall with scattering of the same rock boulders without direct sunlight. The area covered within these images is more than 5×5 m${}^{2}$, however, the actual surface area used by the proposed system is limited by the FOV of the sensor suite. Although these sites do not exactly replicate the surface textures of Mars, the main focus of this analysis is computational load, accuracy and fidelity of the proposed technique rather than classification, surface characterisation or object detection. Moreover current and previous planetary robotic missions (such as Mars) have not produced any datasets with LIDAR information or any other sources of active laser-based depth sensing.

The following statistical measures of performance have been chosen for evaluation purposes:

(1) $$\mathrm{Data}\;\mathrm{Reduction}\;\mathrm{Ratio}\;\left(\mathrm{DRR}\right)=\frac{\mathrm{Size}\;(\mathrm{in}\;\mathrm{bytes})\;\mathrm{of}\;\mathrm{mesh}\;\mathrm{at}\;\mathrm{given}\;\delta }{\mathrm{Size}\;(\mathrm{in}\;\mathrm{bytes})\;\mathrm{of}\;\mathrm{full}\;\mathrm{quality}\;\mathrm{mesh}}$$

Given that each triangle structure is 3×32 bit unsigned integers (12 bytes) and the vertex structure is 5×32 bit floating points (i.e., x, y, z, intensity and the measurement error) resulting in 20 bytes.

(2) Size in bytes=(α×12)+(β×20)+8bytes

where, *α* and *β* are the cardinalities of the triangles and vertices respectively.

Given a point *P* and a surface *S*, the geometric deviation $G_{d}$(*P*,*S*) between *P* and *S* is defined as:

(3) $$G_{d}\left(P,S\right)=\min_{P_{s}\in S}\;d\left(P,P_{s}\right)$$

where $d(P,P_{s})$ is the Euclidean distance between the two points *P* and $P_{s}$. The nearest point $P_{s}$ may lie on the edge of a triangle, a vertex or within the bounded area of the triangle. The geometric deviation does not directly measure the difference between two surfaces, just between a point and a surface. However, if the geometric deviation is calculated between every vertex of one mesh and the surface of another mesh; aggregate measures such as the average value, or frequency distribution may also be calculated.

The mesh simplification algorithm proposed in this paper has been tested in the laboratory and in several more relevant outdoor environments. Two different aspects of this algorithm are evaluated here; the computing resources used to create and store the produced mesh and its geometric quality. At each site a fusion scan was conducted using the rover, and the resulting raw scan data was converted into a simplified textured mesh surface 50 times. Each mesh was created at a different *δ* ranging from 0 m to 0.1 m in 0.002 m increments. This allowed the performance of the algorithm and the effects of different levels of mesh simplification to be accurately compared by using the same raw data.

**Figure 20:**
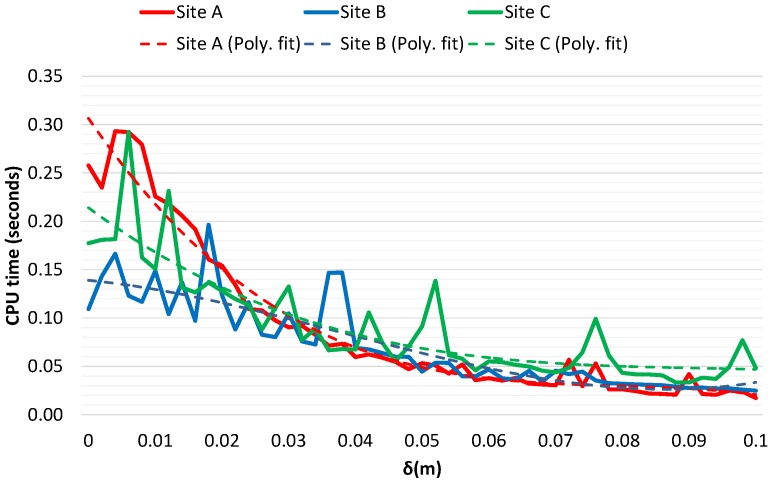
CPU time taken to create mesh at different *δ* values approximated by a third order polynomial trendline to reduce data fluctuation.

The results for CPU usage in Figure 20 plots the time taken to generate the 3-D surface at different values of *δ*. Although there is measurement noise in the measured time, it shows a strong correlation with the amount of memory used by each mesh. To overcome the fluctuation a third order polynomial trendline is overlapped onto the original data. The trend in these plot manifest the speed of the algorithm to generate simplified surfaces from the raw LIDAR data. This is in direct contrast to traditional mesh simplification algorithms that generally have incremental execution time relative to the amount of mesh simplification required.

**Figure 21:**
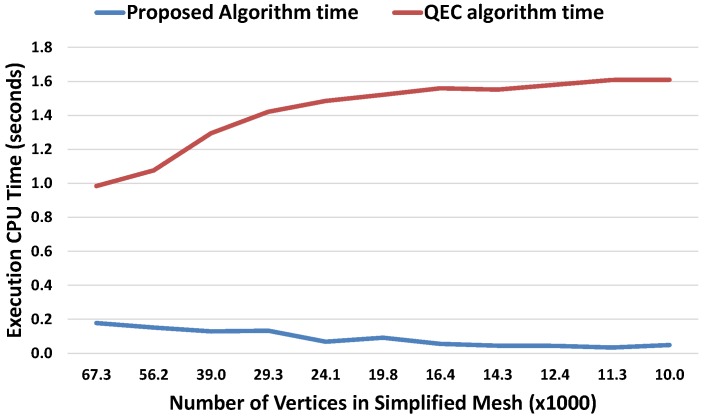
A comparison of the execution time of the proposed algorithm versus the QEC technique.

The Quadratic Edge Contraction (QEC) developed by Michael Garland and Paul Heckbert [52] algorithm was chosen as a representative existing solution to mesh simplification. A comparison of the execution time of the proposed algorithm was performed against the QEC technique, as illustrated in Figure 21. Although the comparative analysis does not perform a like-for-like comparison since the two algorithms specify the target quality differently, i.e., proposed technique uses the minimum distance between vertices (*δ*) and QEC uses the target number of triangles. However in this experiment the QEC algorithm was executed following the proposed algorithm with the target number of triangles set to the number of triangles produced by the proposed technique. It is worth noting that the proposed algorithm executes almost an order of magnitude faster than QEC and positively correlates with the produced mesh quality, unlike QEC which correlates negatively.

**Figure 22:**
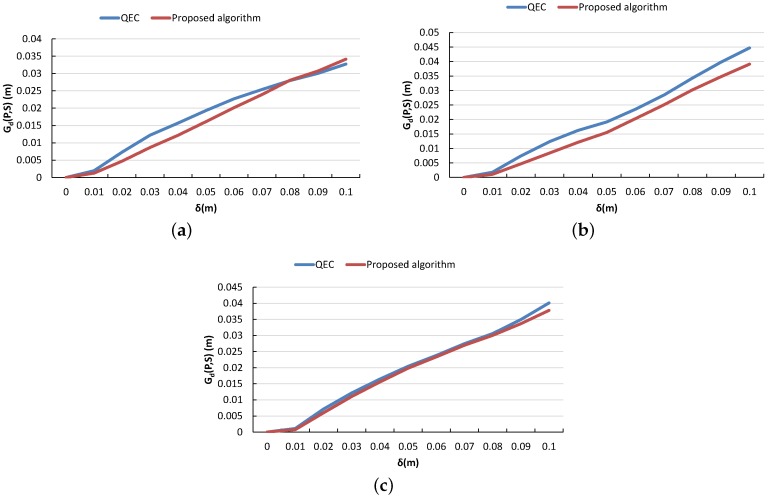
A comparison of the proposed algorithm versus the QEC technique in terms of $G_{d}\left(P,S\right)$ for Site A (**a**); Site B (**b**) and Site C (**c**).

The geometric quality of the simplified surfaces produced by the proposed algorithm was compared to QEC algorithm. Since the level of simplification required is defined differently by both algorithms; the plots shown in Figure 22 were generated as follows. The full quality mesh was first simplified using the proposed algorithm at the given delta value and the cardinality of faces was measured. Next the full quality mesh was simplified using the QEC technique such that the cardinality of the faces was equal to that of the proposed algorithm. The geometric deviation between the full quality mesh and the two simplified meshes was then calculated. This process was repeated for a range of delta values from 0 to 10 cm at each of the three test sites. Referring to the plots in Figure 22; the geometric deviation from the full quality mesh is greater for the QEC technique as compared to the proposed algorithm. However further investigation will be required in future to seek the exact nature of difference in the geometric qualities of these two algorithms.

**Figure 23:**
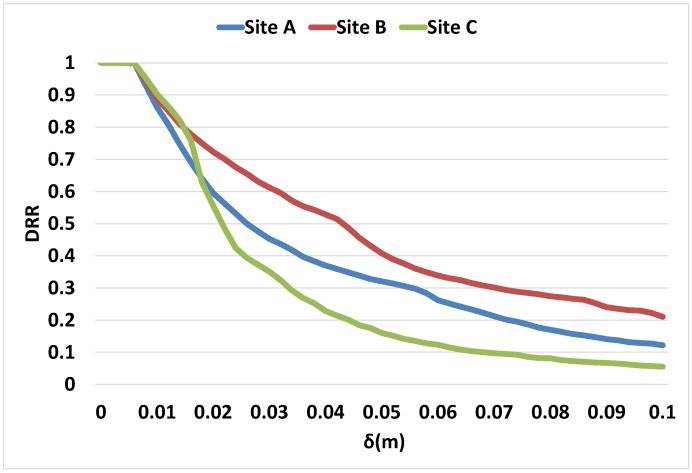
Data reduction ratio with change in *δ* values.

There is a very significant reduction in the amount of memory required to store the mesh data as the *δ* value is increased. Figure 23 plots the variation in the DRR of the three different sites used. The greatest reduction can be seen in the scan at site C. This mesh is on average closer to the sensor compared to the other two scans, therefore the raw LIDAR samples have much finer detail which is more quickly decimated with the increase of *δ*. The site facing a building on the other hand contains a large number of samples close to the maximum range of the LIDAR, therefore the *δ* value must get above the vertex spacing in this area before this part of the mesh begins to decimate. Finally, site A has an even range of depths from near to the maximum range of the sensor, therefore the DRR of this mesh lies between that of the other two sites.

**Figure 24:**
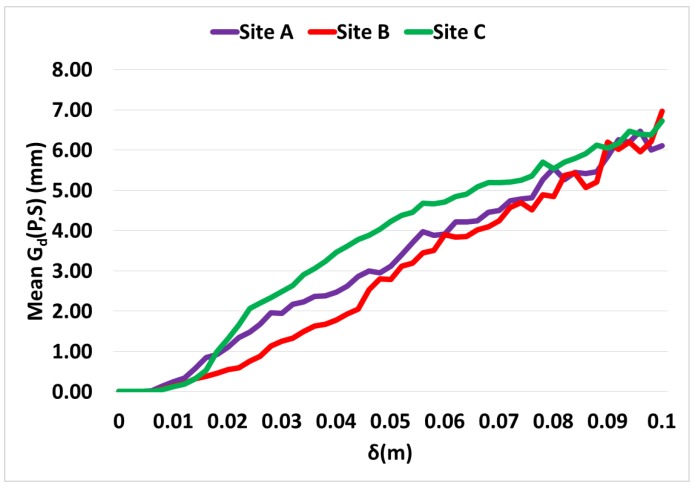
Mean geometric deviation (mm) for different *δ* values.

Figure 24 shows the increase in the mean geometric deviation ($G_{d}\left(P,S\right)$) with the increase in *δ*. If the plots in Figure 23 are compared with Figure 24, it can be clearly noticed that there is a significant reduction in the size of the mesh even when the mean geometric deviation is low. In the case of site C; the point where the mesh has reduced by half in size, the mean geometric deviation is less than 2 mm. Similarly, the mean geometric deviation is around 2 mm for site B, when its mesh size has reduced by half as well. These results show that the algorithm is performing very well since a large reduction in mesh size and CPU time can be achieved without compromising the geometric quality of the mesh in a way that may affect the usefulness of the data.

**Figure 25:**
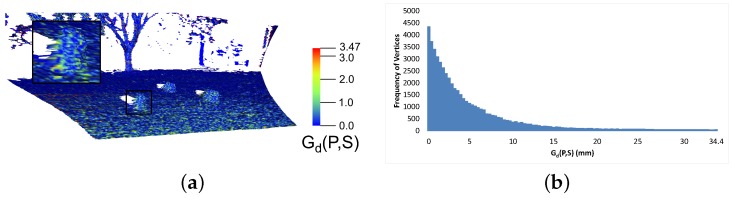
Surface plot of $G_{d}\left(P,S\right)$ for an outdoor environment (**a**) and the respective histogram (**b**).

Mesh generated at a *δ* value of 0.06 m is illustrated in Figure 25. There is some geometric deviation across the whole mesh. The histogram shows the majority of deviations are below 0.01 m.

## 4. Discussion

This paper presented an in-depth review of the various visual perception sensors and techniques used on board planetary rovers for scene reconstruction and terrain modelling. The popular stereo vision-based technique used for previous and contemporary rover missions was discussed along with its limitations that may deem it infeasible for future planetary rover missions. To overcome stereo-vision limitations, techniques for 3-D reconstruction of the terrain from monocular images were introduced. However, most of these techniques have been tested for specific terrestrial applications using limited data, and may require further investigation and optimisation for planetary rover applications. Light Detection And Ranging (LIDAR) technology has proven capabilities for precise long and short target detection, identification, and depth estimation on board orbital spacecrafts and terrestrial robotic navigation. Therefore, it was introduced as a potential sensor for future planetary missions. Due to specific complexities within the point cloud data from LIDAR as well as the absence of texture information, the current research work proposed the fusion of camera information with LIDAR data to overcome some of these limitations and make it more suitable for planetary rovers.

A thorough investigation into the fusion of camera with LIDAR data was presented such that the resulting solution is potentially feasible for planetary rover GNC systems. A number of challenges were mentioned that are related to data from LIDAR sensors. LIDAR point clouds in combination with high resolution camera images may produce large amounts of data. Therefore, the proposed solution is optimised such that the lowest possible computational and memory resources are utilised. The technique was further examined using statistical metrics to identify its potential for planetary rovers. Experimental results in Section 3.7 manifests low computation load, very high accuracy and fidelity. The technique was examined using three different sites with varying topological and environmental constraints.

## 5. Conclusions

The research findings in this paper clearly show a transformation in the field of depth perception and 3-D surface reconstruction for planetary rovers, not just in terms of the hardware sensors, but also in terms of state-of-the art methods for multi-sensor fusion to extend the capabilities of the planetary rovers for future missions. These techniques however require further validation, verification and testing on space-qualified LIDARs and processing units using highly optimised algorithms, calibrated sensors and datasets that replicate the Martian surface characteristics.
